# Temperature-Caused Changes in Raman Pattern and Protein Profiles of Winter Triticale (x *Triticosecale*, Wittm.) Field-Grown Seedlings

**DOI:** 10.3390/molecules29091933

**Published:** 2024-04-24

**Authors:** Iwona Stawoska, Aleksandra Wesełucha-Birczyńska, Gabriela Golebiowska-Paluch

**Affiliations:** 1 Institute of Biology and Earth Sciences, University of the National Education Commission, Krakow, Podchorążych 2, 30-084 Kraków, Poland; iwona.stawoska@up.krakow.pl; 2 Faculty of Chemistry, Jagiellonian University, Gronostajowa 2, 30-387 Kraków, Poland; birczyns@chemia.uj.edu.pl

**Keywords:** wintering, cold hardening, Raman spectroscopy, photosynthetic dyes, catalase, peroxidase, superoxide dismutase, peroxiredoxin, photosystem II proteins

## Abstract

Climate change, which causes periods with relatively high temperatures in winter in Poland, can lead to a shortening or interruption of the cold hardening of crops. Previous research indicates that cold acclimation is of key importance in the process of acquiring cereal tolerance to stress factors. The objective of this work was to verify the hypothesis that both natural temperature fluctuations and the plant genotype influence the content of metabolites as well as proteins, including antioxidant enzymes and photosystem proteins. The research material involved four winter triticale genotypes, differing in their tolerance to stress under controlled conditions. The values of chlorophyll *a* fluorescence parameters and antioxidant activity were measured in their seedlings. Subsequently, the contribution of selected proteins was verified using specific antibodies. In parallel, the profiling of the contents of chlorophylls, carotenoids, phenolic compounds, and proteins was carried out by Raman spectroscopy. The obtained results indicate that a better PSII performance along with a higher photosystem II proteins content and thioredoxin reductase abundance were accompanied by a higher antioxidant activity in the field-grown triticale seedlings. The Raman studies showed that the cold hardening led to a variation in photosynthetic dyes and an increase in the phenolic to carotenoids ratio in all DH lines.

## 1. Introduction

The successful wintering of cereals and the quality and quantity of their yield are largely influenced by tolerance to many stress factors at the same time. These include tolerance to abiotic factors, which act below or above the optimum value for an individual plant genotype and development stage, such as temperature and access to water, as well as biotic factors such as infection with fungal pathogens. As shown by many years of research [1,2,3,4,5,6,7], low temperatures may cause an increase in cereal tolerance to stresses that coexist in winter, which could remain persistent during the following early spring period and even in the plant’s adult stage. In contrast, cold de-acclimation connected with climate warming could adversely affect winter hardiness [6,7].

Triticale (x *Triticosecale* Wittm.) is a synthetic hybrid derived by crossing between wheat (*Triticum aestivum* L., AABBDD) and rye (*Secale cereale* L., RR); consequently, it contains both wheat (AABB or AABBDD) and rye (RR) genomes, whereas the D genome is fully eliminated during the breeding process [8]. Currently cultivated, hexaploid triticale (AABBRR) is well-adapted to adverse environmental conditions: low temperatures [2,9,10,11,12,13,14,15], aluminum toxicity [16], drought [1], salinity [17], and waterlogged soils [18]. All of these qualities make triticale a very promising cereal and a valuable genetic source for transferring desirable genes, in particular stress-tolerance genes from rye to wheat [8].

In a recent Rapacz et al. [19] study performed on empirical models of winter wheat and triticale, different weather variables affected the winter survival of both species. Winter wheat was affected by winter severity, the number of freezing–thawing cycles, the thermal vegetation index, and the freezing index in various winter months, while triticale was affected by late winter ice encasement and high numbers of freeze–thaw events. In Rys et al.’s [20] study, de-acclimation resulted in a decreased winter oilseed rape frost tolerance that was accompanied by a decrease in the content of sugars and an increase in the osmotic potential. The chemical composition of the leaves measured using FT-Raman spectroscopy also clearly confirmed the metabolic differences between the cold-acclimated and de-acclimated plants [20].

The photosynthetic electron transport (PET) chain and photosystem II (PSII) are involved in the induction of defense-related genes, as well as cold- and light-related genes [5]. Chlorophyll *a* fluorescence has been reported as being a sensitive indicator of stress effects on physiological and biochemical changes in the photosynthetic apparatus [21,22]. Chlorophyll *a* fluorescence data might be analyzed using the JIP test, which is based on the theory of energy flow in thylakoid membranes [22]. The proteins of the reaction center of photosystem II and the proteins that bind to this center have multi-pass helical thylakoid transmembrane chains [22].

In previous research results (e.g., Fv/Fm, ABS/Csm, Tro/CS, and Eto/CS), correlations were shown with a genotypically dependent level of cold-induced tolerance of triticale to stress factors such as drought, low temperatures, and fungal infections [1,2,3,5]. Other important physiological indicators included leaf water status and phenolic acids content [1], as well as proline and sugars content [23]. Lipid peroxidation level, measured as thiobarbituric acid reactive substances (TBARS) content, and the activity of antioxidant enzymes such as catalases, peroxidases, and superoxide dismutases in seedlings of Hewo and Magnat parent cultivars and offspring DH lines have been used as biomarkers in previous studies, which revealed genotypically dependent changes as a result of triticale cold acclimation under controlled conditions [9,11].

The Raman profiles of winter triticale cold-chamber-tested seedlings were also differentiated under the influence of temperature changes [14]. Raman spectroscopy was established as an alternative to other expensive and time-consuming techniques (e.g., chromatography). Usually, it does not require sample preparation or solvent extraction before analysis, hence, it has found applications in biological, biochemical, biomedical, and agricultural sciences for tissues, grains, fluids, and whole cells [24,25,26]. The mentioned technique allows for the simultaneous examination of various biomolecules belonging to sugars, photosynthetic dyes, lipids, or proteins and the evaluation of molecular changes occurring in tested samples under biotic and abiotic stress factors, infections, fertilization treatments, or other agricultural applications [27,28,29]. Raman spectroscopy is based on the phenomenon of inelastic light scattering by molecules that are being excited to a higher vibrational state. Tested samples can be treated with monochromatic UV, Vis, or NIR light that is emitted from a laser. The light causes the vibration of the molecules and, as a consequence, characteristic bands typical for selected chemical structures appear on the Raman spectrum. The intensity of the obtained signal can be also used for quantitative calculations of the relative concentrations of selected compounds in the sample. Furthermore, a mathematical analysis (e.g., example spectrum deconvolution) can provide answers regarding the participation of individual protein secondary structures, the conformation of SS bridges, or the coordination state in metal complexes [25,28,30,31]. However, as with other experimental methods, this one has also its limitations. One of the most important is the autofluorescence of the sample, which appears when visible radiation is used. In this way, the fluorescence signal is so strong that it often obscures the actual Raman signals of the substance. A solution for this problem is a spectrometer with a laser with a wavelength of 1064 nm (near-IR excitation wavelength), which was also used in our measurements. Decreasing the energy of the laser radiation allowed for the fluorescence signal to be reduced and, consequently, such a procedure makes the identification of substances easier in the examined sample.

In turn, the technique of gel electrophoresis combined with electrotransfer and immunostaining allows for the quantitative and qualitative analysis of the individual protein abundance in the tested samples, and then a comparison of the protein profiles between the objects. In previous studies, antioxidant enzymes, in particular peroxidases, Glutathione-S-Transferases, and thioperoxiredoxins, were identified by a proteomic approach as being involved in maintaining the balance of oxido-reduction processes during winter triticale, barley cold acclimation, and tolerance to the following stresses [11,12,14,32,33]. In winter barley seedlings, highly differentiated in terms of freezing tolerance, changes in protein abundance related to photosynthesis, carbohydrate metabolism, oxidation-reduction reactions, and stress response were observed under the influence of cold hardening. The above results of proteomic studies are complemented by the evaluation of photosynthetic apparatus acclimation, showing distinctive differences between the studied genotypes in the number of active PSII reaction centers (RC/CSm). In winter barley DH lines with a high freezing tolerance, hydrogen peroxide generation during cold-hardening treatment was accompanied by more stable catalase activity [32].

Another factor of great importance was the ability to sustain, during drought stress, the relatively high activity of enzymes [33]. In silico, identified candidate genes related to triticale cold acclimation, as well as following freezing and drought tolerance, included the wheat and rye genes involved in chloroplast RNA processing, chromatin organization, the regulation of gene expression, serotonin, and very-long-chain fatty acids’ biosynthesis [34].

None of the published studies included the physiological and biochemical profiling of triticale exposed to the natural temperature course with the set of methods presented here. The present work extended existing findings with the use of selected winter triticale lines from the doubled haploid (DH) cvs. Hewo x Magnat mapping population, which was previously derived and tested for drought [1], *Microdochium nivale* infection [2], and freezing [3] tolerance in the seedling stage after cold treatment in a cool chamber, as well as for *Blumeria graminis* [4] field tolerance in the adult stage. For these stress factors, the genotype-dependent immunity after the seedlings’ cold acclimation was observed in our previous study, and the genome regions, as well as candidate genes, proteins, and other biomolecules associated with the cold treatment and resulting stress tolerance level, were identified [34].

The analyses performed here were used to test the research hypothesis about the influence of the plant genotype and cultivation conditions on the content of metabolites as well as proteins, including antioxidant enzymes and proteins related to the photosystem II. Additionally, it was hypothesized that genotypes with a higher photosynthetic efficiency have higher antioxidant activity, reflecting the more efficient oxiredox balance. The obtained data will be useful for future functional research under cold/de-acclimation conditions in crop plants and as markers for the stress tolerance testing of cereal genotypes.

## 2. Results

### 2.1. Field Cultivation Conditions

The soil analysis results, taken before the plant material was sown, are summarized in Tables S1–S3. In soil samples, the optimum pH values were measured. The content of the available microelements was in the average range and did not require soil supplementation. Also, the N_min_ content in kg/ha in the 0–60 cm layer was medium. Phosphorus fertilization was recommended not to be used. The scales (ranges) of the contents of soil components were given by the District Chemical and Agricultural Station in Krakow (No AB 759). Due to these data, no fertilizers or chemical protection products were used throughout the experiment duration.

The values of average daily air temperature [°C], minimum daily ground temperature [°C], average daily rainfall [mm], and average daily snow cover [cm] are presented in Figure S1. The first cultivation period (28 September–2 November 2022) lasted 35 days. The range of observed temperatures was between +6 °C and +14 °C, while the median and average temperature value was +11 °C ± 2.2 °C. Therefore, this cultivation period was defined as the seedling pre-hardening period. The second cultivation period (3 November 2022–13 January 2023) lasted 71 days. The range of observed temperatures was between −10 °C and +10 °C, while the average and median temperature value was +3 °C ± 4.1 °C. Thus, this cultivation period was specified as the seedling cold-hardening period I. The third cultivation period (14 January–31 January 2023) lasted 17 days. The range of observed temperatures was between −2 °C and +5 °C, while the median and average temperature was +1 °C ± 1.8 °C. Thus, this cultivation period was assigned as the seedling cold-hardening period II.

The average temperature for the autumn/winter observation period between 28 September 2022 and 31 January 2023 (125 days in total, approx. 18 weeks) was +5 °C ± 5.3 °C. In turn, the average temperature for the entire observation period between 28 September 2022 and 31 August 2023 (337 days in total, approx. 48 weeks/11 months) was +10 °C ± 7.9 °C. The minimal experimental air (−11 °C) and ground (−22 °C) temperatures were observed on 19 December 2022.

### 2.2. Analysis of Chlorophyll a Fluorescence

In the results obtained via a stationary fluorimeter for three measurement dates (2 November 2022, 13 January, and 26 January 2023), significant effects of the seedling genotype, cultivation conditions, and the interaction of these factors (genotype × conditions) on the Fv/Fm values were observed according to the ANOVA test (Table 1). The plant genotype, as well as the interaction of the genotype with the cultivation conditions, also influenced the NPQ values (Table 1).

Moreover, the significant correlation coefficient with the plant genotype was noted for the Fv/Fm, Qymax, and NPQ values, according to Kendall’s Tau test (Table S4). The cultivation conditions were correlated with the Rfd values (Table S4). The interaction of two independent factors, genotype × conditions, was correlated with the Fv/Fm and NPQ values (Table S4).

**Figure 1:**
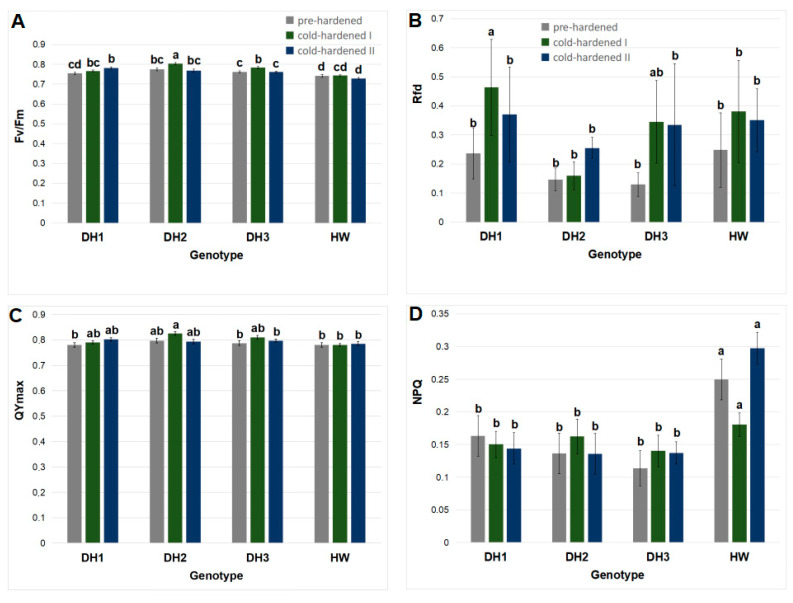
Values of chlorophyll *a* fluorescence parameters: (**A**) Fv/Fm; (**B**) Rfd; (**C**) QYmax; and (**D**) NPQ measured using stationary fluorimeter in field grown winter triticale seedlings of the DH1–3 lines and the parental cultivar Hewo (HW), subjected to pre-hardening (control), as well as the first (I) and second (II) cold-hardening periods. The bars present average values ± SD (n = 20); the letters indicate the similarities or differences between the compared groups in terms of the value of the dependent variable, according to the Kruskal–Wallis test.

Based on the above chlorophyll *a* fluorescence results, it can be assumed that the Fv/Fm values were influenced by the seedling genotype (Table 1 and Table S4), cultivation conditions (Table 1), and interaction of these factors (Table 1 and Table S4). The maximal experimental mean value of this parameter was observed for the DH2 line in the second cultivation period (cold-hardening period I), while minimal values were noted for cv. Hewo during all cultivation periods (Figure 1A). The seedlings of the DH1 line showed an Fv/Fm increase after exposure to the cold-hardening period II. In contrast, in the seedlings of the remaining two lines, DH2 and DH3, the Fv/Fm increase was noted earlier, after the cold-hardening period I. In the cv. Hewo seedlings, the Fv/Fm values did not show significant changes during the entire winter observation period, unlike previous cold chamber test results [2].

In the present research, the Rfd values were correlated only with the cultivation conditions (Table S4); the highest values were observed in the DH1 seedlings after the cold-hardening period I (Figure 1B). The QYmax values were correlated only with the seedling genotype (Table S4); the maximal and minimal mean values of this parameter were observed under the cold-hardening period I for the DH2 line and cv. Hewo seedlings, accordingly (Figure 1C). The NPQ values were influenced by the seedling genotype as well as the interaction of this genotype with the cultivation conditions (Table 1 and Table S4); the maximal values were observed in the cv. Hewo seedlings during the entire winter cultivation period (Figure 1D).

**Figure 2:**
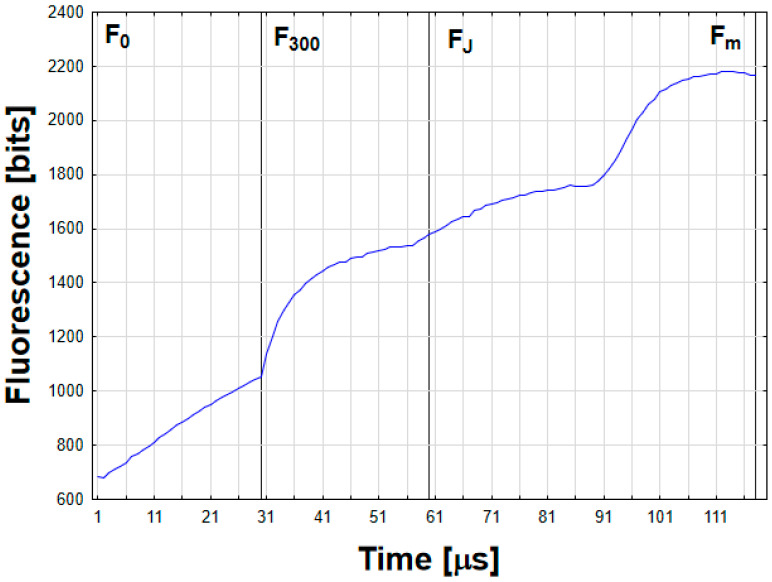
The example chlorophyll fluorescence induction curve, recorded for triticale seedling leaf. Time-points used for OJIP test parameters calculation are indicated.

In previous research, the chlorophyll *a* fluorescence parameters of Fv/Fm, ABS/Csm, Tro/CS, and Eto/CS values were also correlated with a genotypically dependent level of cold-induced tolerance of winter triticale to drought, low temperatures, and fungal infections [1,2,3,5]. A typical chlorophyll *a* fluorescence spectrum obtained in winter triticale seedlings grown in the field, along with the measured parameters, is shown in Figure 2.

### 2.3. Antioxidant Activity

On the basis of the enzymatic assay results obtained for three measurement dates (2 November 2022, 13 January, and 26 January 2023), significant effects of the seedling genotype, cultivation conditions, and interaction of these factors (genotype × conditions) on the Cu/Zn SOD I activity were observed (Table 1). Both genotype (Table 1) and cultivation conditions (Table 1 and Table S4) had effects on the Fe/Mn SOD activity. The activity of CAT, POX, and Cu/Zn SOD II was correlated only with the cultivation conditions (Table S4).

**Figure 3:**
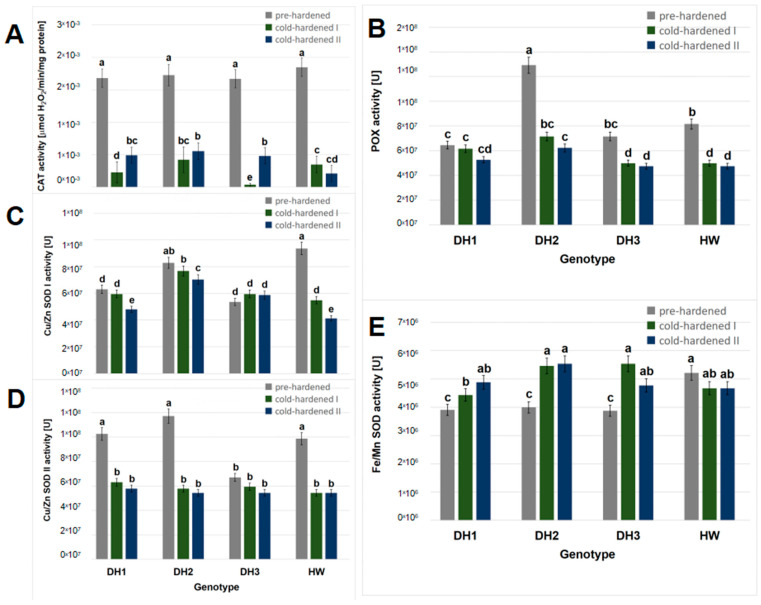
Antioxidant enzymes activity: (**A**) total catalase; (**B**) total peroxidase; (**C**) Cu/Zn SOD I; (**D**) Cu/Zn SOD II; and (**E**) Fe/Mn SOD activity in field-grown winter triticale seedlings of the DH1–3 lines and the parental cultivar Hewo (HW), subjected to pre-hardening (control), as well as the first (I) and second (II) cold-hardening periods. The bars present average values ± SD (n = 4); the letters indicate the similarities or differences between the compared groups in terms of the value of the dependent variable, according to the Kruskal–Wallis test.

In pre-hardened seedlings, the maximum mean CAT activity was observed for all genotypes. In turn, after the cold-hardening period I, the CAT activity was the highest in the seedlings of the DH2 line. The remaining genotypes presented decreasing CAT activity, in the order of: cv. Hewo > DH1 > DH3. After the cold-hardening period II, the CAT activity was similar in the seedlings of the DH lines, while it was the lowest in the seedlings of cv. Hewo (Figure 3A). Also, in previous studies, differential inductions of CAT activity were found between resistant and susceptible genotypes of winter triticale. In non-hardened seedlings of the tolerant DH1 line, the CAT activity was lower compared to the values obtained for the susceptible line, but the enzyme activity was enhanced by cold treatment only in the resistant genotype [11].

In pre-hardened seedlings field-cultivated in the presented work, the maximum mean POX activity was observed for the DH2 line, while the lowest was detected in the DH1 seedlings. In turn, after the cold-hardening period I, the POX activity decreased in all genotypes with the exception of the DH1 line and was, again, the highest in the seedlings of the DH2 line. The lowest activity of this enzyme was observed in the DH3 and cv. Hewo seedlings. After the cold-hardening period II, the POX activity remained on the same level as that noted during the cold-hardening I period (Figure 3B). Under controlled conditions, the total POX activity increased in the DH1 seedlings after exposure to low temperatures in relation to the non-hardened control of this genotype, as well as in comparison to a non-tolerant DH line [11].

Before cold hardening, in the present investigation, the maximum mean Cu/Zn SOD I activity was observed for the cv. Hewo and DH2 seedlings, while the lowest activity was observed in the DH1 and DH3 lines. After the cold-hardening period I, the Cu/Zn SOD I activity decreased in the seedlings of cv. Hewo, but remained the highest in the DH2 seedlings. At the end of the cold-hardening period II, it decreased again in the seedlings of all genotypes with the exception of the DH3 line with stable activity; it remained the highest in the DH2 seedlings (Figure 3C). Before cold hardening, the lowest mean Cu/Zn SOD II activity was observed in the DH3 line. In the seedlings of the remaining genotypes, it was similar and decreased after the cold-hardening period I (Figure 3D). In previous studies conducted under controlled conditions, the Cu/Zn SOD I and II activity increased prominently in DH1 seedlings after exposure to low temperatures in relation to the non-hardened control of this genotype [11].

In pre-hardened seedlings, the maximum mean Fe/Mn SOD activity was observed in the present research for cv. Hewo. After the cold-hardening period I, the Fe/Mn SOD activity increased only in the seedlings of the DH lines to the level observed in cv. Hewo during the entire cultivation period (Figure 3E). In contrast, previously, no MnSOD activity was detected in non-hardened plants by native electrophoresis [11]. However, as in the present results, exposure to low temperatures led to an increase in MnSOD activity in DH1 seedlings [11].

### 2.4. Immunodetection of Proteins

A positive reaction was obtained between the antibodies used and protein epitopes in the tested extracts of winter triticale seedlings (Figure S2). On the basis of the results obtained for three measurement dates (2 November 2022, 13 January, and 26 January 2023), significant effects of the seedling genotype, cultivation conditions, and interaction of these factors (genotype × conditions) on the PrxQ abundance were observed (Table 1). Additionally, the PsbC and PsbD abundances were correlated with the cultivation conditions (Table S4).

**Figure 4:**
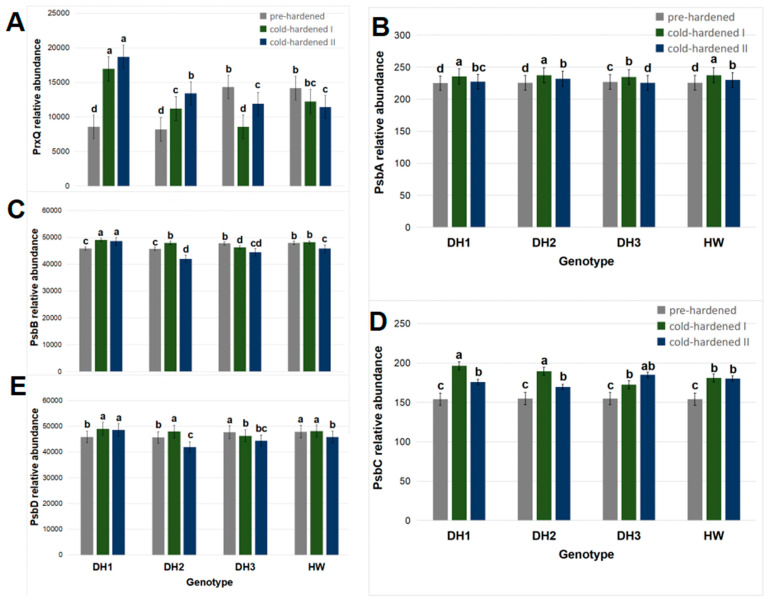
Protein relative abundance: (**A**) thioredoxin reductase PrxQ; (**B**) protein D1 (PsbA) of photosystem II; (**C**) protein CP47 (PsbB) of photosystem II; (**D**) protein CP43 (PsbC) of photosystem II; and (**E**) protein D2 (PsbD) of photosystem II in field-grown winter triticale seedlings of the DH1–3 lines and the parental cultivar Hewo (HW), subjected to pre-hardening (control,) as well as the first (I) and second (II) cold-hardening periods. The bars present average values ± SD (n = 4); the letters indicate the similarities or differences between the compared groups in terms of the value of the dependent variable, according to the Kruskal–Wallis test.

The mean relative abundance of the PrxQ protein was higher in the cv. Hewo pre-hardened seedlings compared to the seedlings of the DH lines on the same date (Figure 4A).

After the cold-hardening period I, the PrxQ abundance decreased in the DH3 seedlings in comparison to the pre-hardened control of this line, but after the cold-hardening period II, it returned to the level observed in cv. Hewo. In contrast, in the cv. Hewo seedlings, the PrxQ abundance was stable after the cold-hardening period I, but after the cold-hardening period II, it decreased in comparison to the pre-hardened plants of this genotype. For the remaining two lines—DH1 and DH2—an increase in the PrxQ content was observed after both cold-hardening periods I and II compared to the pre-hardened plants of these lines. Moreover, the maximal experimental values of this parameter were observed in seedlings of the DH1 line after exposure to both cold-hardening periods I and II (Figure 4A). Under controlled conditions, in cv. Hewo seedlings analyzed using 2D SDS PAGE protein profiling, a cold-mediated accumulation of peroxiredoxin content was observed [12]. Similarly, in studies conducted on the influence of temperature on seedlings of the DH1 line in controlled conditions, increases in the relative expression of the gene encoding peroxiredoxin and the relative content of this protein were observed, estimated by the qRT-PCR and LC-MS techniques, respectively [10,14].

In the present research, the mean relative abundance of the PsbA protein was similar in the investigated genotypes except for the DH3 line, in which it was higher. In all seedlings, after the cold-hardening period I, the PsbA abundance increased when compared with the pre-hardened control, and after the cold-hardening period II, it decreased. After this period, it was the lowest in the seedlings of the DH3 line. The remaining genotypes showed similar changes in PsbA content: an increase after the cold-hardening period I in relation to pre-hardened plants, as well as a decrease after the cold-hardening period II in relation to the cold-hardening period I to a level higher than that observed in the pre-hardened plants of these lines. Moreover, the maximal experimental values of this parameter were observed in the seedlings of the DH1, DH2 lines, and cv. Hewo after exposure to the cold-hardening period I (Figure 4B).

In pre-hardened seedlings, the mean relative abundance of the PsbB protein was higher in the DH3 and cv. Hewo than in the DH1 and DH2 lines. In the cv. Hewo seedlings, after the cold-hardening period I, the PsbB abundance was stable in comparison to the pre-hardened control of these genotypes. In contrast, it decreased after the cold-hardening period II. In the DH2 seedlings, the PsbB abundance increased after the cold-hardening period I and then decreased after the cold-hardening period II. In the DH3 seedlings, the PsbB abundance showed a progressive decline after the cold-hardening period I and cold-hardening period II. In contrast, in the seedlings of the DH1 line, the PsbB abundance increased after the cold-hardening period I and remained maximal also after the cold-hardening period II (Figure 4C).

The mean relative PsbC protein abundance was similar in the pre-hardened seedlings of all genotypes. After the cold-hardening period I, it increased in all seedlings, and was the highest in the DH1 and DH2 lines. After the second cold-hardening period, the PsbC content was again similar in the seedlings of all genotypes (Figure 4D).

After pre-hardening, the mean relative abundance of the PsbD protein was higher in the DH3 and cv. Hewo seedlings. Only in the DH1 and DH2 seedlings did the PsbD abundance increase after the cold-hardening period I in comparison to the pre-hardened controls. After the cold-hardening period II, it remained stable in the DH1 seedlings, while it decreased in all the remaining seedlings (Figure 4E).

### 2.5. FT-Raman Studies

**Figure 5:**
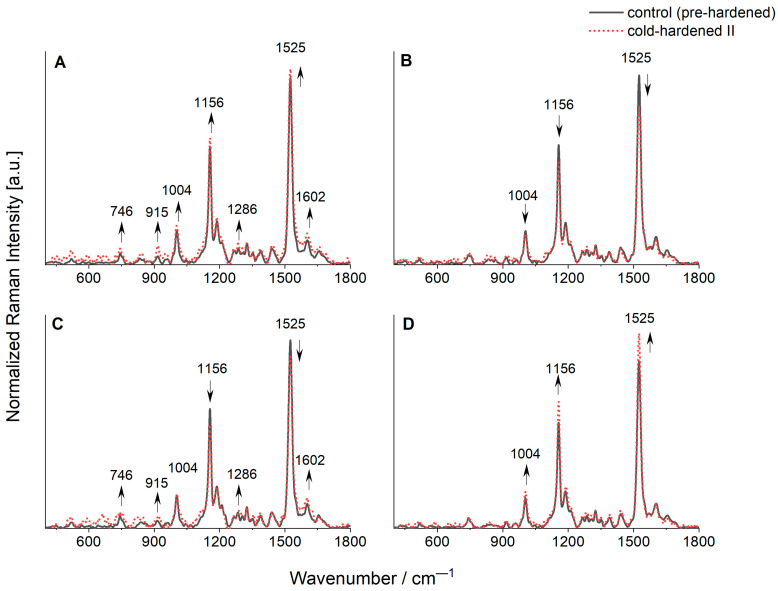
FT-Raman spectra, normalized according to 1325 cm^−1^ (pyrrole ring breathing vibrations), obtained for lyophilized leaves of triticale genotypes growing in a natural field condition: (**A**) DH1 line, (**B**) DH2 line, (**C**) DH3 line, and (**D**) HW cultivar. Solid black line—spectra obtained for control leaves before the cold-hardening period (pre-hardened); Dotted red lines—spectra obtained after the cold-hardening period II. Particular spectra represent the average values of at least 3–6 replications.

Based on the normalized and average spectra obtained from the FT-Raman measurements recorded on lyophilized leaves of various genotypes of triticale, namely the DH1, DH2, and DH3 lines, as well as cv. Hewo (HW), the vibrational bands assigned to the main types of biomolecules, mainly chlorophylls and carotenoids, phenolic compounds, and proteins were characterized (Figure 5).

Typical bands for carotenoids called the carotenoid tripled and identified at 1004, 1156, and 1525 cm^−1^ were observed. The most intensive one, localized at 1525 cm^−1^, originates from the stretching modes of conjugated C=C in the central chain. According to the literature data, the localization of this band is connected with the number of conjugated C=C bonds, and the position identified in the obtained spectra is typical for nine conjugated bonds in the fluorescent molecules that are found in β-carotene, lutein, and other dyes from xanthophyll-cycle [35,36]. This fact causes the band with the maximum at 1525 cm^−1^ to be related to the total content of carotenoids in the tested plant samples. However, it is worth adding that carotenoid side groups, as well as their interactions with other biologically active molecules, can influence the discussed band wavenumber [35,36]. The band found at 1156 cm^−1^ is attributed to the stretching modes of single C-C bonds in the carotenoid central chain, whereas the least intense one at 1004 cm^−1^ is typical for C-CH_3_ rocking modes [30,37,38].

An increase in the Raman bands’ intensity after the cold-hardening period II was found for HW and a slight increase in the intensity of the carotenoid triplet was also recorded for the DH1 genotype (Figure 5A,D). The opposite effect was observed for DH2 and DH3, which may suggest partial degradation of the mentioned dyes in these two latter genotypes (Figure 5B,C).

Weak bands at 1354, 1286, 915, and 746 cm^−1^, together with shoulders at 1550 and 987 cm^−1^, are attributed to chlorophylls [37,39,40,41]. For DH1 and, to a very limited extent, for DH3 genotypes, intensity increases of these bands after the cold-hardening period II were observed. What is especially interesting is that, after the cold-hardening period II, an additional weak band appears at the app. 1570 cm^−1^ for the DH1 and DH3 lines (Figure 5A,C). According to Pascal et al., this band is also connected to chlorophylls, however, it rather belongs to chlorophyll *b* than chlorophyll *a* [42].

Phenols are the most abundant secondary metabolites present in plants, which constitute a very diverse group of chemical compounds. These compounds can be divided into phenolic acids, derivatives of phenolic acids, and flavonoids. Typically, they comprise an extensive variety of molecules that have at least one hydroxyl group (-OH) bonded directly with an aromatic hydrocarbon structure. The Raman spectroscopy technique is used to identify and characterize both pure substances and mixed compounds. The contribution of phenolic compounds in the tested plant samples was observed at 1602 cm^−1^. The band localized at this Raman shift is the most typical and usually the most intensive found for phenolic compounds. For the DH1 and DH3 genotypes, a slight increase in the band intensity was seen after the cold-hardening period II (Figure 5A,C). One of the chemical defense mechanisms of plants is the production of phenolic compounds that protect against herbivore attacks, but also against pathogens and many abiotic stress features. Regarding these, the increase in the phenolic compounds may be connected with a protection role and a selected genotype response to weather and temperature conditions.

**Figure 6:**
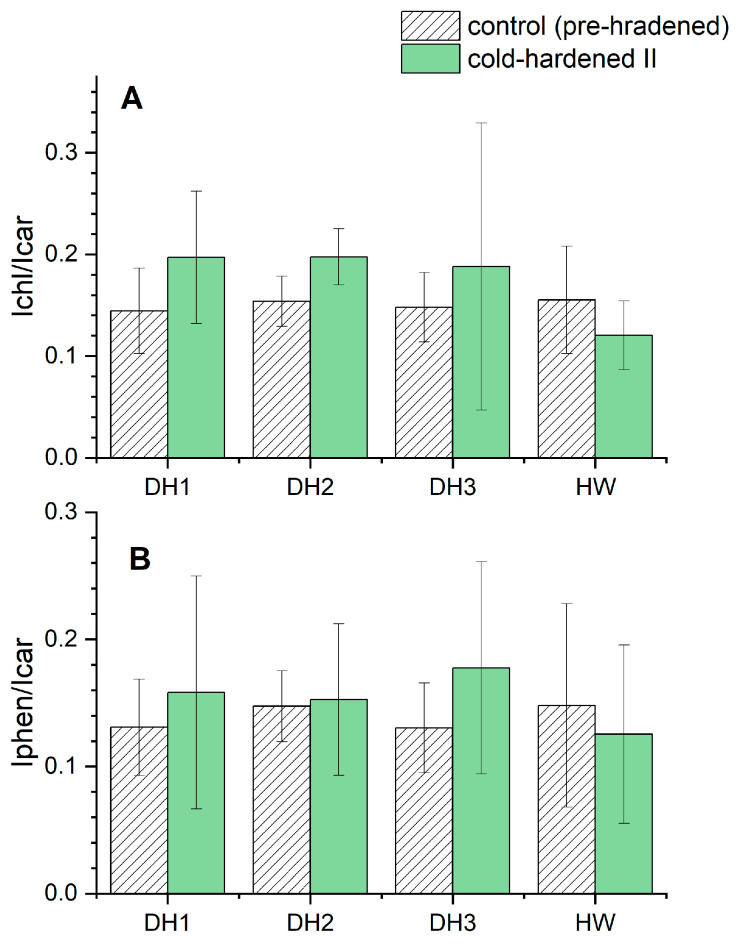
Ratios (±s.d.) of intensities of bands of: (**A**) chlorophyll (1550 cm^−1^) to carotenoid (1525 cm^−1^) contents (Ichl/Icar) and (**B**) phenolic compounds (1602 cm^−1^) to carotenoid (1525 cm^−1^) contents (Iphen/Icar), estimated on the basis of FT-Raman spectra obtained for the cold-hardening period II (green bars) and control leaves of seedlings (grey bars) belonging to DH1, DH2, and DH3 lines and HW cultivar of triticale; n ≥ 4.

The minor increase of the amide I band at 1658 cm^−1^ (typical for protein structures) after the cold-hardening period II was observed only for DH1, whereas for DH2 and HW, a decrease was observed, and for DH3, no visible changes were detected (Figure 5). These results suggest that low temperatures have an impact on the production/degradation of proteins, however, this depends on the genotype of triticale. Changes were also observed in the signal intensity ratio of chlorophyll dyes versus carotenoids Ichls/Icar (Figure 6A). For ratio calculations, the chlorophyll band located at 1550 cm^−1^ was chosen, which is assigned to central 16-membered ring vibrations and the C-C stretching of the pyrrole ring [43], and the most intense band (at 1525 cm^−1^) from carotene triplet. An increase in the chlorophylls to carotenoids ratio after the cold-hardening period II was observed for the DH1, DH2, and DH3 lines (whereas for the HW genotype, the opposite result was seen). Therefore, there must have been a change in the content of chlorophylls to carotenoids to the detriment of the latter. The same tendency for DH1, DH2, DH3, and HW was seen for a ratio of phenolic compounds to carotenoids, Iphen/Icar, where the used band (and its intensity) for phenols was detected at 1602 cm^−1^ (Figure 6B). However, the smallest effect of weather conditions was noticed for the DH2 line (the ratio of Iphen to Icar) and the most visible changes were recorded for the DH3 genotype. As was already pointed out above, the plant response in the form of numerous phenolic compounds is known to be related to various stress factors. One of the most important is UV radiation, where epidermal phenolics act as screening compounds and play a complementary role as protectors under oxidative stress.

It should be also noted that, for the HW genotype of both ratios, namely Ichl/Icar and Iphen/Icar, the opposite effect compared to the DH lines was observed. To be precise, a decrease in both ratios after the cold-hardening period II was detected.

### 2.6. Correlation between the Values of Measured Parameters

In the investigated seedlings, the Fv/Fm and QYmax values were negatively correlated and the NPQ values were positively correlated with the increasing genotype number (in the order of DH1–3 lines and cv. Hewo). This relationship reoccurred between the Fv/Fm and NPQ values and the interaction of the genotype with the cultivation conditions. The Rfd values were negatively correlated with the CAT, POX, and Cu/Zn SOD I activity, as well as positively correlated with the PsbC and PsbD protein abundances. Moreover, the QYmax values were positively correlated with the PsbA, PsbC, and PsbD protein abundances, while the Fv/Fm values only with the PsbC abundance (Table S4).

Among the tested antioxidant enzymes, only the Cu/ZnSOD II activity was correlated with the interaction of the genotype with the cultivation conditions. The activity of the remaining enzymes was correlated only with the cultivation conditions. The CAT activity was positively correlated with POX, as well as the Cu/Zn SOD I and II activity. The activities of these four enzymes were negatively correlated with the PsbA, PsbC, and PsbD protein abundances. In contrast, the Fe/Mn SOD activity was positively correlated with the abundances of the above proteins (Table S4).

The abundance of PrxQ was positively correlated with the PsbB content. The PsbA abundance was positively correlated with the content of the remaining PSII proteins. The abundance of PsbC was positively correlated with the PsbD content; the contents of both these proteins were positively correlated with the cultivar conditions (Table S4).

### 2.7. Location of Coding Genes and Protein Conformation

Based on the amino acid sequences identified in our previous studies [14], the location of the coding genes for the specific proteins described in this work was in silico located, both in the wheat (chr1D, chr2A-D, chr3A-D, chr4A-B, chr5D, chr6A, and D, as well as chr7A and D) and rye genomes (chr2R-7R). The highest number of candidate genes was found on chromosomes 2A-D and 2R (Table S5). According to an Alphafold analysis, thioredoxin with cysteine sulfenic acid (-SOH) intermediate as an active site was located in the cytosol, while the superoxide dismutase copper/zinc binding domain was in the chloroplast and the manganese/iron superoxide dismutase N/C-terminal domain was in the mitochondrion. In turn, multi-pass thylakoid membrane PsbA-D proteins had 2–7 helical transmembrane chains (Table S6).

## 3. Discussion

The above studies aimed to answer the question of whether the same mechanisms and molecules that have been described for winter triticale seedling tolerance to dehydration caused by frost and drought under controlled conditions are responsible for tolerance under natural temperature conditions in the field.

Based on the analysis of the course of temperature values during the experiment, the autumn/winter observation period was divided into three periods: pre-hardening (also as a control for further periods), cold-hardening period I, and cold-hardening period II. Neither fertilizers nor chemical protection products were used in the entire experiment, therefore, the presented results refer only to the influence of natural conditions occurring in field cultivation.

The analysis of chlorophyll *a* fluorescence confirmed that the PSII efficiency (Fv/Fm) was influenced by the seedling genotype, cultivation conditions, and interaction of these factors. In parental cv. Hewo seedlings, the Fv/Fm values did not show changes during the entire winter observation period, and simultaneously, were minimal for the entire experiment. In contrast, the maximal experimental value of this parameter was observed for the DH2 line after the cold-hardening period I. In the seedlings of the remaining two lines, DH1 and DH3, an Fv/Fm cold-mediated increase was also noted. Moreover, the highest Rfd values were observed for the DH1 seedlings after both cold-hardening periods I and II. During the cold-hardening period II, the maximal and minimal QYmax values were observed for the DH2 line. Based on the results obtained, it can be assumed that, in the DH1 and DH2 lines, PSII adapts more efficiently to changing temperature conditions, including cold periods and freeze–thawing cycles.

After the cold-hardening period II, CAT activity, which protects cells from the toxic effects of hydrogen peroxide, was higher in the seedlings of the DH lines than in cv. Hewo. After the cold-hardening period II, the POX, Cu/Zn SOD I, and Fe/Zn SOD activity was the highest in the seedlings of the DH2 line. On this basis, it can be assumed that the better PSII performance and higher contents of D1 and D2 PSII proteins were accompanied by a higher level of antioxidative activity in the field-grown triticale seedlings. The D1/D2 (PsbA/PsbD) reaction center heterodimer binds P680, chlorophylls that are the primary electron donors of PSII, and subsequent electron acceptors. It shares a non-heme iron and each subunit binds pheophytin, quinone, additional chlorophylls, carotenoids, and lipids. D1 provides most of the ligands for the Mn4-Ca-O5 cluster of the oxygen-evolving complex (OEC). There is also a Cl-ion associated with D1 and D2, which is required for oxygen evolution. The PSII complex binds additional chlorophylls, carotenoids, and specific lipids [44].

The protein and Raman profiles of genotypes which varied in terms of their physiological status in the field conditions were further analyzed and compared for the identification of candidate biomolecules involved in the response to these new stresses. In contrast to the CAT activity, the peroxiredoxin/thioredoxin reductase (PrxQ) abundance was positively correlated with the PsbB abundance. Without cold hardening, the PrxQ abundance was higher in the DH3 and cv. Hewo seedlings in comparison to the DH1 and DH2 lines on the same date. However, after the cold-hardening period I, the PrxQ abundance decreased in the DH3 and cv. Hewo seedlings. In contrast, for the remaining two lines—DH1 and DH2—a constant, cold-mediated increase in the PrxQ content was observed. Moreover, the maximal experimental values of this parameter were observed in seedlings of the DH1 line after exposure to both cold-hardening periods I and II. It can be assumed that, in the DH1 and DH2 lines PrxQ may have a protective function against oxidative stress in triticale seedlings, since this chloroplastic thiol-specific peroxidase (EC:1.11.1.24) catalyzes the reduction in hydrogen peroxide and organic hydroperoxides to water and alcohols, respectively, as well as plays a role in cell protection against oxidative stress by detoxifying peroxides [45].

After the cold exposure period II, the maximum experimental values of the PsbA and PsbB abundances were observed in the seedlings of the DH2 and DH1 lines, accordingly. Moreover, the PsbA abundance was positively correlated with the PsbB and PsbD abundances. PsbB (CP47 reaction center protein) is one of the components of the core complex of PSII that binds chlorophyll and helps to catalyze primary light-induced photochemical processes [46]. In our study, the PsbD content increased as a result of cold hardening in the seedlings of all tested genotypes, but only in the DH1 and DH2 seedlings did we obtain the maximum experimental values of this parameter. Both the DH1 and DH2 lines had the highest PsbC abundance after cold-hardening period II, as well as the highest PsbD abundance after cold-hardening period I.

The Raman studies showed that the cold-hardening period II influenced the ratio of photosynthetic dyes (chlorophylls versus carotenoids), and for all DH genotypes, led to a decrease in carotenoids content in favor of the content of chlorophyll pigments. Furthermore, for the DH1 and DH3 lines, an additional band typical for chlorophyll *b* (chl *b*) was observed. That suggests a possible influence of the cold-hardening period II on the formation of chl *b* in plastids. Results concerning the interactions of chlorophyll molecules in spinach antenna protein presented by Pascal et al. [42] suggest that this band is also sensitive to the coordination state and its localization at 1570 cm^−1^ suggests that the central Mg atom of chl *b* molecules is 5-coordinated. The cold-hardening period II also had an impact on the phenolic to carotenoids ratio, and for all DH genotypes, an increase in the ratio was observed. This fact suggests that an increasing content of phenolic compounds compared to carotenoid dyes can be connected with a protective role of the former against stress factors.

In conclusion, the first tests showed differentiation in the physiological and biochemical parameters for selected winter triticale genotypes grown under natural cold-hardening conditions. Two of these lines reached the highest values of the measured parameters after being exposed to low temperatures (marked as the DH1 and DH2 lines). To our knowledge, these are the first extensive studies on the impact of the natural cold-hardening period on the biochemical profiles in winter triticale. Further analysis could lead to a description of the mechanisms and diagnosis of markers of field tolerance of winter triticale to different temperature regimes.

## 4. Materials and Methods

### 4.1. Plant Material and Experimental Design

In the present study, three winter triticale doubled haploid (DH) lines selected from the mapping population derived by the anther method [47] from an F1 hybrid of cv. ‘Hewo’ (Strzelce Plant Breeding-IHAR Group Ltd., Strzelce, Poland) and cv. ‘Magnat’ (DANKO Plant Breeders Ltd., Kościan, Poland) were cultured in field conditions along with parental cv. ‘Hewo’. The DH lines were selected based on multi-year tolerance tests under controlled conditions, as described by Gołębiowska et al. [2], according to increasing seedling susceptibility to snow mold (P index), sequentially: DH1 (38), DH2 (47), cv. Hewo (58), and DH3 (69). Additionally, the most different snow mold tolerance DH lines were also tolerant (DH1 and DH2) or susceptible (DH3) to freezing under controlled conditions [3]. The selected genotypes were also characterized by different levels of powdery mildew *Blumeria graminis* (DC.) Speer. tolerance in the generative phase in field cultivation [4]; cv. Hewo and DH1 plants had the lowest (2.7 and 5.7), DH3 plants had intermediate (6.7), and DH2 plants had the highest (7.5) mean degree of infection examined in 17 experiments, where 1 refers to an immune plant and 9 to a susceptible one.

Healthy and well-formed kernels of the above genotypes were sown before the end of September on the experimental plots with coordinates 50°10′09.6′′ N 19°54′42.1′′ E. The seeds were planted directly into the ground in a randomized block of replicates. Throughout the period from seedling to harvest, they were exposed to naturally occurring conditions.

The weather parameters during the entire breeding period (28 September 2022–31 August 2023) were collected from the nearest weather stations (station codes: 350190566 and 250190390; station names: Kraków-Balice and Kraków-Obserwatorium) at https://danepubliczne.imgw.pl/data/dane_pomiarowo_obserwacyjne/, accessed on 1 September 2023. The following daily parameters were recorded: minimum and maximum air temperature [°C], average air temperature [°C], minimum ground temperature [°C], ground condition [W/R], total precipitation [mm] and its duration [h], type of precipitation, total rainfall [mm], snow fall [mm] and snow cover thickness [cm], snow water equivalent [mm/cm], average humidity [%], average wind speed [m/s], duration of wind ≥10 m/s [h], average cloud cover [octants], and daily sunshine [h]. Additionally, the durations of fog, mist, soot, ice, storm, dew, and frost were recorded in hours. Soil pH and the contents of micro- and macroelements were measured at the District Chemical and Agricultural Station in Krakow, Poland (Accreditation Certificate of Testing Laboratory No AB 759).

### 4.2. Sampling

Samples were taken in replicates in terms on 2 November 2022 (control/pre-hardening period), as well as on 13 January 2023 (the cold-hardening period I), 26 January, and 31 January 2023 (the cold-hardening period II) for the analysis of chlorophyll a fluorescence, FT Raman spectroscopic measurements, and sample freezing at −80 °C for proteomic analysis.

### 4.3. Analysis of Chlorophyll a Fluorescence

Seedling intact leaves were analyzed in 20 biological replicates for 4 triticale genotypes in the first, second, and third breeding periods. The JIP test parameters were evaluated according to Rapacz et al. [22] using a stationary FluorCam 700 ST 664-009656 fluorometer FMS 2 (Photon Systems Instruments, 664 24 Drásov, Czech Republic) for freshly cut leaves on 2 November 2022 (pre-hardening period), as well as 13 January 2023 (the cold-hardening period I) and 26 January (cold-hardening period II). According to Maxwell and Johnson [48], actinic light was used for chlorophyll fluorescence excitation in PSII and the steady-state fluorescence yield (Fs) was stabilized. The maximal fluorescence yield (Fm) was measured in leaves that were dark-adapted for 20 min. Based on the fluorescence spectra, the JIP test parameters were calculated: (1) specific energy fluxes for single PSII reaction centers (RCs): absorbed energy flux (ABS/RC), trapped energy flux (TRo/RC), electron transport flux (ETo/RC), and dissipated energy flux (DIo/RC); and (2) phenomenological energy fluxes calculated for the area of the photosynthetic sample (CS) at t = 0: absorbed energy flux per CS (ABS/CS), trapped energy flux per CS (TRo/CS), electron transport flux per CS (ETo/CS), and dissipated energy flux per CS (DIo/CS). The calculated PSII performance indexes and reaction center densities included a performance index calculated on an absorption basis (PIABS) and the densities of QA-reducing PSII reaction centers at t = 0 and tmax (time to reach maximum fluorescence Fm), corresponding to RC/CSo and RC/CSm, respectively. The maximum quantum yield of primary photochemistry at t = 0 (Fv/Fm) was calculated, where Fv—variable fluorescence yield.

### 4.4. Enzymatic Assays

All measurements of antioxidant enzyme activity were performed in at least 4 biological and 4 instrumental replicates. For the extraction of CAT and SOD, 0.1 g of plant material was homogenized in liquid nitrogen and re-suspended in cold phosphate buffer (0.1 mol·dm^−3^ KH_2_PO_4_/Na_2_HPO_4_ pH 7.5, containing 3 mmol·dm^−3^ ethylenediaminetetraacetic acid EDTA and 2% (*w*/*v*) polyvinylpolypyrrolidone) in a proportion of 3:1 v/FW. For the extraction of POX, 0.1 g of plant material was homogenized in liquid nitrogen and re-suspended in cold 50 mmol·dm^−3^ sodium acetate buffer at pH 5.5 in a proportion of 3:1 v/FW. The homogenate was centrifuged at 12,000× *g* for 5 min at 4 °C, and the supernatant was used for measuring the antioxidant enzyme activity, according to Bergmeyer [49]. The SOD activity was assayed as described in detail by Golebiowska and Gawronska [11]. The protein concentration was determined according to the Bradford method [50].

The CAT activity was determined according to Aebi [51]. The reaction mixture contained 50 mmol·dm^−3^ potassium phosphate buffer at pH 7.0, 0.1 mmol·dm^−3^ EDTA, 0.04% (*v*/*v*) H_2_O_2_, and 20 µL of enzyme extract in a 1 mL total volume. The decomposition of H_2_O_2_ was measured at 240 nm per 1 min. As a unit of enzyme activity, a decrease in absorbance equal to 0.0145 was assumed (consumption of 1 µM of H_2_O_2_). The extinction coefficient of H_2_O_2_ of 42.6 mol^−1^·dm^3^ cm^−1^ was used. The spectroscopic analysis was performed using an Ultrospec 2100 pro UV/Visible spectrophotometer (GE Healthcare, UK Limited, Warszawa, Poland).

### 4.5. Western Blot

Verification of the presence and relative content of the selected proteins was carried out in 4 biological replicates by the immunoblotting technique, according to the modified protocol provided by the Bio-Rad company for three measurement dates (2 November 2022, 13 January, and 26 January 2023). The extracted proteins were subjected to a protein concentration evaluation by the Bradford [50] method. Aliquots of 5 µg protein samples were loaded in triplicates onto 12% polyacrylamide separating gels overlayed by 4% stacking gel along with Prestained Western Blotting Protein Standards (Bio-Rad, Kraków, Poland). One-dimensional denaturing SDS electrophoresis was performed in 10 mM Tris-HCl (pH 8.0) with 0.1 mM EDTA (TE) running buffer in replicates in a Mini-PROTEAN^®^ Tetra System electrophoresis chamber (Bio-Rad) at RT for 15 min at 24 mA/gel, followed by 30 min at 40 mA/gel. After separation, the gels were used to create blotting sandwiches with ready-to-use Trans-Blot Turbo Mini 0.2 µm PVDF Transfer Packs #1704156 (Bio-Rad). The electrotransfer of proteins onto the membrane was performed in a Trans-Blot^®^ TurboTM Transfer System (Bio-Rad) at 25 V/1.3 A. After 7 min, the gels were removed, and membranes were picked for immunostaining. Before taking the next steps, the top of the first protein lane was marked on the membrane by cutting off a small fragment. Then, the membranes were incubated (25 mL/membrane) in Tris Buffered Saline (TBS) with 1% casein blocking solution (Bio-Rad). After washing three times in Tris-buffered saline with 0.1% Tween^®^ 20 Detergent (TBST) for 20 min, the membranes were incubated overnight in 25 mL of the primary antibody in TBST solution. The following primary antibodies were used: (1) polyclonal against Peroxiredoxin, thioredoxin reductase PrxQ (Q9LU86, At3g26060) of expected MW 16 kDa (AS05 093, Agisera, Vännäs, Sweden); (2) polyclonal against PsbA/D1 protein of PSII, C-terminal (thylakoid membrane marker) of expected MW 28–38 kDa (AS05 084, Agisera); (3) polyclonal against PsbB/CP47 protein of PSII of expected MW 56 kDa (AS04 038, Agrisera); (4) polyclonal against PsbC/CP43 protein of PSII of expected MW 43–45 kDa (AS11 1787, Agrisera); and (5) polyclonal against PsbD/D2 protein of PSII of expected MW 28–39 kDa (AS06 146, Agrisera) in (1) 1: 5000; (2) 1: 10,000; (3) 1: 2000; (4) 1: 3000; and (5) 1: 10,000 dilutions, respectively. Next, three 20 min washes in TBST were followed by 2 h of incubation in 25 mL of solution of a secondary polyclonal antibody conjugated with the detecting system, which allowed for the visualization of specifically recognized proteins (Rabbit anti-Goat IgG (H+L) Secondary Antibody, AP, Invitrogen, Warszawa, Poland). After one short wash in TBST, the membranes were incubated for 2 min in darkness in a 5 mL/membrane solution of 5-bromo-4-chloro-3-indolyl-phosphate in conjunction with Nitro Blue Tetrazolium (BCIP/NBT Color Development ready to use Solution, Bio-Rad). Then, the staining solution was decanted from above the membranes and allowed to dry and decolorize the background. The received images were scanned in the ChemiDoc MP System (Bio-Rad) and analyzed with Image J software/Lab Program ver 4.1 (Bio-Rad).

### 4.6. FT-Raman Spectroscopy

The Raman spectra of the lyophilized leaves of non-stressed and stressed seedlings of the cultivar Hewo (HW), as well as the DH1, DH2, and DH3 winter triticale lines, were measured as performed previously [14] with a Thermo Scientific Nicolet NXR 9650 FT-Raman spectrometer (Madison, WI, USA) equipped with a InGaAs (indium gallium arsenide) detector and a Micro-Stage Microscope. The samples were excited with a 1064 nm line of the Nd:YAG laser, since this excitation wavelength minimizes the effect of autofluorescence from the sample. The following experimental parameters were used for all collected spectra: 200 mW laser power, accumulation from 500 scans, and measurement in the range of 4000–300 cm^−1^. For each measured leaf (three leaves for each type of triticale genotype, both stressed for the cold-hardening period II and control), at least three spectra on both its adaxial and abaxial sides were performed. The obtained spectra were averaged using spectra obtained from at least three different spatial points of each analyzed sample, and then baseline correction (then subtraction) of the background by the second derivative mode with 18 points and β-spline connection was used. This procedure allowed for the extraction of Raman signals from the registered results containing the fluorescence background contribution (the intrinsic fluorescence of the samples). In the next step, a normalization procedure at 1325 cm^−1^ was employed. The wavelength was chosen as a reference, since it is typical for pyrrole ring breathing vibrations that are observed for all tested samples (the vibrations of chlorophyll *a* and *b*). Finally, analyses in the range of 2000–300 cm^−1^ were performed and the identification of the vibration characteristics for the selected molecules according to the cited literature data was conducted. The Origin Pro 2020 program was used for all mathematical analysis.

### 4.7. Statistical Analysis

Statistical significance was evaluated by the Shapiro–Wilk normality test followed by a multi-factor analysis of variance ANOVA for data with a normal distribution. Next, the Kendall’s Tau correlation coefficient estimation for all parameters, including categorical (genotype, treatment, and genotype × treatment) and quantitative (parameter values), were calculated. For all data, the Kruskal–Wallis test was performed to indicate the similarities (same letters) or differences (different letters) between the compared groups in terms of the value of the dependent variable. A statistical analysis was performed with STATISTICA^®^ version 13.0 software at *p* ≤ 0.05.

### 4.8. Bioinformatic Analysis

The amino acid sequences of the proteins examined in the presented work were taken from the results of the LC-MS analysis performed for the same genotypes in our previous work [14]. Then, using the Blast tool in GrainGenes service [52], the location of the coding sequences in the wheat and rye reference genomes was estimated, as described in detail by Gołębiowska et al. [2]. Only high-confidence and high-identity (>95%) genes were selected. Next, by using the Alphafold tool [53,54], the same amino acid sequences were used for protein data, including conformation estimation.

## 5. Conclusions

- The analysis of chlorophyll *a* fluorescence confirmed that PSII efficiency (Fv/Fm) was influenced by seedling genotype, cultivation conditions, and the interaction of these factors.
- For DH1 and DH2 lines, PSII adapts more efficiently to changing temperature conditions, including cold periods and freeze–thawing cycles, compared to DH3 and cv. Hewo seedlings. In DH1 and DH2 lines, PrxQ may have a protective function against oxidative stress.
- Both the DH1 and DH2 lines had the highest PsbC abundance after the cold-hardening period II, as well as the highest PsbD abundance after the cold-hardening period I.
- For the DH1 and DH3 lines, after the cold-hardening period II, an additional band typical for chlorophyll *b* (chl *b*) was observed (possible influence of the cold-hardening period II on the formation of chl *b* in plastids)
- An increasing content of phenolic compounds compared to carotenoid dyes after the cold-hardening period II observed for the DH lines can be connected with a protective role of the former against stress factors.

## Figures and Tables

**Table 1:** The level of significance (*p* values obtained by multi-factor ANOVA test) of the independent categorical factors’ impact (genotype, cultivation conditions, and their interaction) on the physiological and biochemical (quantitative) parameters of winter triticale seedlings grown in the field during the (1) control/pre-hardening period, (2) the cold-hardening period I, and (3) the cold-hardening period II.

Parameter		Genotype	Cultivation Conditions 1–3	Genotype × Conditions 1–3
Chlorophyll *a* fluorescence	Fv/Fm	*****	*****	****
	NPQ	*****	ns	*
Enzyme activity	Cu/Zn SOD I	****	***	****
	Fe/Mn SOD	**	**	ns
Protein abundance	PrxQ	***	*	*****

*—*p* < 0.05, **—*p* < 0.01, ***—*p* < 0.005, ****—*p* < 0.001, *****—*p* < 0.0005, and ns—not significant.

## Data Availability

All data generated or analyzed during this study are included in this published article. Moreover, the datasets used and/or analyzed during the current study are available from the corresponding author on reasonable request.

## Supplementary Materials

The following supporting information can be downloaded at: https://www.mdpi.com/article/10.3390/molecules29091933/s1, Table S1. Available forms of minerals in [mg/100 g of soil] or in [mg/kg of soil] determined in the soil. Tests performed at the District Chemical and Agricultural Station, Krakow, Poland (accredited laboratory, PCA AB 759); Table S2. Nitrogen (and its forms) content in soil samples taken at a depth of 0–30 cm and 31–60 cm. Tests performed at the District Chemical and Agricultural Station, Kraków, Poland (accredited laboratory, PCA AB 759); Table S3. pH, salinity and content of selected available nutrients in [mg/dm3] of the soil. Tests performed at the District Chemical and Agricultural Station, Kraków, Poland (accredited laboratory, PCA AB 759). Table S4. Kendall’s Tau correlation coefficients between categorical and quantitative variables. Table S5. List of high confidence and high identity (>95%) genes encoding selected proteins identified in winter triticale DH1–3 seedlings by LC-MS [14] and their physical location on wheat (Wheat Chinese Spring IWGSC RefSeq v2.1 proteins, 2021) and rye (Secale cereale Lo7 v1 pseudomolecules, 2021) reference genomes according the GrainGenes Blast Service. Table S6. Data obtained using AlphaFold for amino acid sequences of selected winter triticale proteins identified in seedlings of winter triticale line DH1–3 in Gołębiowska et al. [14]. Figure S1. Climatic conditions: (A) average daily temperature; (B) daily minimum ground temperature; (C) daily rainfall and (D) daily snow cover depth, measured by the Kraków-Obserwatorium (red line) and Kraków-Balice (blue line) meteorological stations during the winter triticale field cultivation period from 28 September 2022 to 31 August 2023. Figure S2. The reference immunostained blot membrane with visible PrxQ bands approx. 28 kDa.
