# Supplementary material for: Temperature-Caused Changes in Raman Pattern and Protein Profiles of Winter Triticale (x Triticosecale, Wittm.) Field-Grown Seedlings
Source: Molecules. 2024 Apr 24;29(9):1933. doi: 10.3390/molecules29091933 (PMC11085197; doi:10.3390/molecules29091933)
Supplement: Supplementary file 1 [file molecules-29-01933-s001.zip › molecules-2921648-supplementary.pdf]

molecules

MDPI

Article

Temperature-caused changes in Raman pattern and protein profiles of winter triticale (x *Triticosecale*, Wittm.) field-grown seedlings

Table 1S. Available forms of minerals in [mg/100g of soil] or in [mg/kg of soil] determined in the soil. Tests performed at the District Chemical and Agricultural Station, Krakow, Poland (accredited laboratory, PCA AB 759).

**Laboratory code of the sample**	**Agronomic category of soil**	**pH**	**Need for liming**	**Available forms of minerals [mg/100g of soil]**			**Available forms of minerals [mg/kg of soil]**				
				**Phosphorus**	**Potassium**	**Magnesium**	**Boron**	**Manganese**	**Copper**	**Zinc**	**Iron**
				**P_2O_5**	**K_2O**	**Mg**	**B**	**Mn**	**Cu**	**Zn**	**Fe**
GI/223/1	heavy	6.9	limited	42.0	20.0	13.2	2.35	194.0	6.8	35.5	1064
Abundance				very high	average	high	average				

Table 2S. Nitrogen (and its forms) content in soil samples taken at a depth of 0-30 cm and 31-60 cm. Tests performed at the District Chemical and Agricultural Station, Kraków, Poland (accredited laboratory, PCA AB 759).

**Laboratory code of the sample**	**Dry weight of soil [%]** *	**Depth of the soil layer [cm]**	**Nitrogen content in mg/kg FW of soil** **		**Nitrogen content in mg/kg DW of soil** **		**N_{min} content [kg/ha]** ***	**N_{min} content in 0-60 cm depth of the soil layer [kg/ha]** ***	**Abundance**
			nitrate(V) nitrogen	ammonium nitrogen	nitrate (V) nitrogen	ammonium nitrogen			
			**$N\text{-}NO_3^-$**	**$N\text{-}NH_4^+$**	**$N\text{-}NO_3^-$**	**$N\text{-}NH_4^+$**			
GI/223/1	78.50	0-30	5.58	1.77	7.11	2.25	36.50	78.30	average
GI/223/2	77.70	30-60	6.39	1.95	8.22	2.51	41.80		

* according to PN-ISO 11465:1999; weight method

** according to PB36(ed.3) 03.03.2020 r.; flow colorimetry method

*** according to counting

FW – fresh weight

DW – dry weight

Table 3S. pH, salinity and content of selected available nutrients in [mg/dm^3] of the soil. Tests performed at the District Chemical and Agricultural Station, Kraków, Poland (accredited laboratory, PCA AB 759).

**Laboratory code of the sample**	**pH in H_2O**	**Salinity NaCl [g/dm^3]**	**Available forms of minerals in soil [mg/dm^3 of soil]**						**[mg/dm^3 of soil]**
			**N-NO_3^-**	**N-NH_4^+**	**P**	**K**	**Ca**	**Mg**	**Cl^-**
GI/223/1	7.42	<0.10	<14	19	112	125	1160	150	<27
Method	potentiometry	conductometry	potentiometry		colorimetry	flame photometry		FAAS	potentiometry

Table 4S. Kendall's Tau correlation coefficients between categorical and quantitative variables.

Variable	Kendall's Tau correlation coefficients																
	Geno-type	Treat-ment	Genotype/ Treatment	Fv/Fm	Rfd	QYmax	NPQ	CAT	POX	Cu/Zn SOD I	Cu/Zn SOD II	Fe/Mn SOD	PrxQ	PsbA	PsbB	PsbC	PsbD
**Genotype**		0.02	0.91*	-0.33*	-0.08	-0.19*	0.22*	-0.08	-0.17	-0.07	-0.18	0.17	-0.07	0.06	0.04	0.00	-0.01
**Treatment**	0.02		0.23*	0.10	0.19*	0.10	-0.12	-0.38*	-0.37*	-0.16	-0.39*	0.31*	0.18	0.22	-0.07	0.48*	0.49*
**Genotype/ Treatment**	0.91*	0.23*		-0.28*	-0.02	-0.14	0.19*	-0.17	-0.22	-0.08	-0.24*	0.23	-0.03	0.10	-0.01	0.10	0.10
**Fv/Fm**	-0.33*	0.10	-0.28*		0.04	0.70*	-0.36*	-0.12	-0.15	0.03	-0.12	0.11	-0.11	0.16	-0.09	0.26*	0.18
**Rfd**	-0.08	0.19*	-0.02	0.04		0.02	0.00	-0.30*	-0.39*	-0.40*	-0.08	0.00	0.04	0.11	0.05	0.36*	0.36*
**QYmax**	-0.19*	0.10	-0.14	0.70*	0.02		-0.09	-0.18	-0.13	0.01	-0.20	0.21	-0.10	0.24*	0.02	0.30*	0.32*
**NPQ**	0.22*	-0.12	0.19*	-0.36*	0.00	-0.09		0.00	0.06	-0.04	0.02	0.05	0.00	0.05	0.08	-0.02	-0.01
**CAT**	-0.08	-0.38*	-0.17	-0.12	-0.30*	-0.18	0.00		0.59*	0.38*	0.50*	-0.18	0.07	-0.45*	-0.14	-0.37*	-0.41*
**POX**	-0.17	-0.37*	-0.22	-0.15	-0.39*	-0.13	0.06	0.59*		0.55*	0.42*	-0.10	0.09	-0.38*	-0.11	-0.42*	-0.41*
**Cu/Zn SOD I**	-0.07	-0.16	-0.08	0.03	-0.40*	0.01	-0.04	0.38*	0.55*		0.36*	0.15	-0.10	-0.32*	-0.21	-0.36*	-0.37*
**Cu/Zn SOD II**	-0.18	-0.39*	-0.24*	-0.12	-0.08	-0.20	0.02	0.50*	0.42*	0.36*		-0.29*	0.00	-0.59*	-0.14	-0.45*	-0.46*
**Fe/Mn SOD**	0.17	0.31*	0.23	0.11	0.00	0.21	0.05	-0.18	-0.10	0.15	-0.29*		0.05	0.25*	-0.03	0.24*	0.25*
**PrxQ**	-0.07	0.18	-0.03	-0.11	0.04	-0.10	0.00	0.07	0.09	-0.10	0.00	0.05		0.03	0.34*	0.10	0.12
**PsbA**	0.06	0.22	0.10	0.16	0.11	0.24*	0.05	-0.45*	-0.38*	-0.32*	-0.59*	0.25*	0.03		0.26*	0.43*	0.46*
**PsbB**	0.04	-0.07	-0.01	-0.09	0.05	0.02	0.08	-0.14	-0.11	-0.21	-0.14	-0.03	0.34*	0.26*		0.08	0.15
**PsbC**	0.00	0.48*	0.10	0.26*	0.36*	0.30*	-0.02	-0.37*	-0.42*	-0.36*	-0.45*	0.24*	0.10	0.43*	0.08		0.89*
**PsbD**	-0.01	0.49*	0.10	0.18	0.36*	0.32*	-0.01	-0.41*	-0.41*	-0.37*	-0.46*	0.25*	0.12	0.46*	0.15	0.89*	

* - significant at $p < 0.05$

Table 5S. List of high confidence and high identity (>95%) genes encoding selected proteins identified in winter triticale DH1-3 seedlings by LC-MS [14] and their physical location on wheat (Wheat Chinese Spring IWGSC RefSeq v2.1 proteins, 2021) and rye (Secale cereale Lo7 v1 pseudomolecules, 2021) reference genomes according the GrainGenes Blast Service.

**Protein[1]**	**Wheat Gene ID[2]**	**Position [kb][2]**	**Rye gene ID[3]**	**Position [kb][3]**
**Peroxiredoxin**	TraesCS2A03G0741400	chr2A:515270296..515273357 (+ strand)	SECCE2Rv1G0102710	chr2R:581072142..581075874 (+ strand)
	TraesCS2A03G0225000	chr2A:66150862..66153887 (- strand)	SECCE2Rv1G0102710	chr2R:581072142..581075874 (+ strand)
	TraesCS2B03G0817900	chr2B:457380068..457383196 (+ strand)	SECCE2Rv1G0073480	chr2R:68350578..68359657 (- strand)
	TraesCS2D03G0683800	chr2D:379676742..379679937 (+ strand)	SECCE2Rv1G0073480	chr2R:68350578..68359657 (- strand)
	TraesCS3B03G0517500	chr3B:264165958..264171143 (- strand)	SECCE7Rv1G0504150	chr7R:720067339..720068031 (- strand)
	TraesCS6A02G169100	chr6A:179123508..179124591 (+ strand)	SECCE3Rv1G0172140	chr3R:285120129..285124830 (- strand)
	TraesCS7D03G0919400	chr7D:506580135..506581528 (- strand)	SECCE6Rv1G0388490	chr6R:137198814..137199494 (+ strand)
**Cu/Zn SOD**	TraesCS2A02G121200	chr2A:75734383..75737830 (+ strand)	SECCE2Rv1G0075100	chr2R:82905483..82907633 (+ strand)
	TraesCS2B02G567600	chr2B:767298166..767302982 (- strand)	SECCE7Rv1G0486250	chr7R:404198541..404209486 (- strand)
	TraesCS2D02G538300	chr2D:622541948..622546884 (- strand)	SECCE2Rv1G0133810	chr2R:898525048..898529898 (- strand)
	TraesCS4B02G243200	chr4B:503339915..503343358 (+ strand)	SECCE2Rv1G0133810	chr2R:898525048..898529898 (- strand)
	TraesCS7A02G292100	chr7A:368707084..368723444 (- strand)	SECCE7Rv1G0486250	chr7R:404198541..404209486 (- strand)
	TraesCS7D02G290700	chr7D:345781077..345792831 (- strand)	SECCE7Rv1G0472770	chr7R:147581269..147586540 (+ strand)
**Fe/Mn SOD**	TraesCS2B02G567600	chr2B:767298166..767302982 (- strand)	SECCE2Rv1G0133810	chr2R:898525048..898529898 (- strand)
	TraesCS2D02G538300	chr2D:622541948..622546884 (- strand)	SECCE2Rv1G0133810	chr2R:898525048..898529898 (- strand)
**Photosystem II D1 protein**	TraesCS1D02G071400	chr1D:55148812..55173915 (+ strand)	SECCE2Rv1G0095930	chr2R:428316423..428317106 (- strand)
	TraesCS1D02G179900	chr1D:256966320..256967410 (- strand)	SECCE2Rv1G0095930	chr2R:428316423..428317106 (- strand)
	TraesCS4A02G153400	chr4A:321125047..321126223 (+ strand)	SECCE6Rv1G0394910	chr6R:310365582..310366643 (- strand)
	TraesCS4B02G159100	chr4B:308295572..308296255 (+ strand)	SECCE6Rv1G0394910	chr6R:310365582..310366643 (- strand)
	TraesCS6D02G079900	chr6D:67114716..67115399 (+ strand)	SECCE6Rv1G0394910	chr6R:310365582..310366643 (- strand)
**Photosystem II D2 protein**	**TraesCS2B02G330700**	**chr2B:483054630..483055691 (+ strand)**	SECCE3Rv1G0191090	chr3R:745941704..745942828 (+ strand)
	TraesCS2D02G572800	chr2D:652673942..652674409 (- strand)	SECCE4Rv1G0266260	chr4R:730397587..730399421 (- strand)
	TraesCS3D02G523300	chr3D:607870700..607871714 (+ strand)	SECCE4Rv1G0266260	chr4R:730397587..730399421 (- strand)

	**TraesCS5D02G007900**	**chr5D:4960287..4960754 (+ strand)**	SECCE4Rv1G0266260	chr4R:730397587..730399421 (- strand)
**Photosystem II CP43 protein**	**TraesCS2A02G073900**	**chr2A:37021615..37021851 (+ strand)**	SECCE4Rv1G0241030	chr4R:448242238..448243105 (- strand)
	**TraesCS2D02G573000**	**chr2D:652672478..652672972 (- strand)**	SECCE5Rv1G0376550	chr5R:872830933..872831169 (- strand)
**Photosystem II CP47 protein**	TraesCS1D02G182600	chr1D:257063901..257064993 (+ strand)	SECCE4Rv1	chr4R:448146966..448147956 (- strand)
	TraesCS3A02G215700	chr3A:404332557..404332949 (+ strand)	SECCE5Rv1G0317190	chr5R:338510048..338511574 (- strand)
	**TraesCS3D02G053400**	**chr3D:21907250..21908670 (+ strand)**	SECCE4Rv1	chr4R:448146966..448147956 (- strand)

1 - Amino acid sequences identified in seedlings of winter triticale lines DH1-3 by using LC-MS in Gołębiowska et al. [14].

2 - Wheat Chinese Spring IWGSC RefSeq v2.1 proteins (2021) genome in GrainGenes Blast Service.

3 - Secale cereale Lo7 v1 pseudomolecules (2021) genome in GrainGenes Blast Service.

Table 6S. Data obtained using AlphaFold for amino acid sequences of selected winter triticale proteins identified in seedlings of winter triticale line DH1-3 in Gołębiowska et al. [14].

Alphafold ID	Protein name according to Alphafold	Gene according Alphafold	Uniprot ID	Structure according to Alphafold			
				Length [AA]	Model status	No of units	Location/domains
A0A3B6C702_WHEAT		Unknown	A0A3B6C702	262	predicted		Cytoplasmic/unlocated thioredoxin and disordered domains, active site: cysteine sulfenic acid (-SOH) intermediate
A0A3B6B0I4_WHEAT		Unknown	A0A3B6B0I4	258			
A0A0C4BJ55_WHEAT	Thioredoxin-dependent peroxiredoxin	CAMPLR22A2D_LOCUS2968	A0A0C4BJ55	258			
A0A3B6DER7_WHEAT		Unknown	A0A3B6DER7	240			
A0A3B6ASU3_WHEAT		Unknown	A0A3B6ASU3	247			
A0A3B6ATP9_WHEAT	AhpC-TSA domain-containing protein	Unknown	A0A3B6ATP9	154			
A0A3B6TIG0_WHEAT	Glutaredoxin-dependent peroxiredoxin	Unknown	A0A3B6TIG0	230			
A0A3B6NPJ5_WHEAT		Unknown	A0A3B6NPJ5	226			
A0A3B6ASA5_WHEAT	Superoxide dismutase [Cu-Zn]	Unknown	A0A3B6ASA5	157	predicted		Chloroplastic superoxide dismutase copper/zinc binding domain
A0A3B6RFK1_WHEAT		Unknown	A0A3B6RFK1	201			
A0A3B6TK25_WHEAT		Unknown	A0A3B6TK25	202			
A0A0G2YAJ8_WHEAT		SOD2	A0A0G2YAJ8	164			
A0A3B6IVD1_WHEAT		Unknown	A0A3B6IVD1	174			
A0A3B6CFS9_WHEAT	Superoxide dismutase [Fe-Mn]	Unknown	A0A3B6CFS9	225	predicted		Mitochondrial manganese/iron superoxide dismutase N/C-terminal domain
Q96185_WHEAT		SOD3.1	Q96185	231			
G1FUU8_WHEAT		SOD	G1FUU8	231			
PSBA_WHEAT	Photosystem II D1 protein	psbA	P12463	353	predicted	monomer	Multi-pass thylakoid membrane protein, 5-7 helical transmembrane chains, binding site: [CaMn4O5] clusters, Mg
A0A5P8KK99_WHEAT		psbA	A0A5P8KK99	354			
S4Z999_WHEAT		psbA	S4Z999	353			
A0A3B5ZT76_WHEAT		Unknown	A0A3B5ZT76	353			

A0A3B6HSP7_WHEAT		Unknown	A0A3B6HSP7	365			of chlorophyll a ChlzD1 and PD1, pheophytin a D1, quinone B, Fe cation
A0A3B6C7P1_WHEAT<br>PSBD_WHEAT<br>B2CHJ7_WHEAT	Photosystem II D2 protein	Unknown<br>psbD<br>psbD	A0A3B6C7P1<br>Q36814<br>B2CHJ7	353	predicted	mono mer	Multi-pass thylakoid membrane protein, 5-6 helical transmembrane chains,<br>binding site: Mg of chlorophyll a ChlzD2 and PD2, pheophytin a D2, plastoquinone Q(A), Fe cations
A0A5P8KMB2_WHEAT<br>S4Z090_WHEAT<br>B2CHJ8_WHEAT	Photosystem II CP43 reaction center protein	psbC<br>psbC<br>psbC	A0A5P8KMB2<br>S4Z090<br>B2CHJ8	483<br>473<br>473	predicted	mono mer	Multi-pass thylakoid membrane protein, 5-6 helical transmembrane chains
A0A3B6GPT0_WHEAT<br>S4Z9D7_WHEAT<br>PSBB_WHEAT	Photosystem II CP47 reaction center protein	Unknown<br>psbB<br>psbB	A0A3B6GPT0<br>S4Z9D7<br>P24065	385<br>508<br>508	predicted	mono mer	Multi-pass thylakoid membrane protein, 2/6 helical transmembrane chains

molecules

MDPI

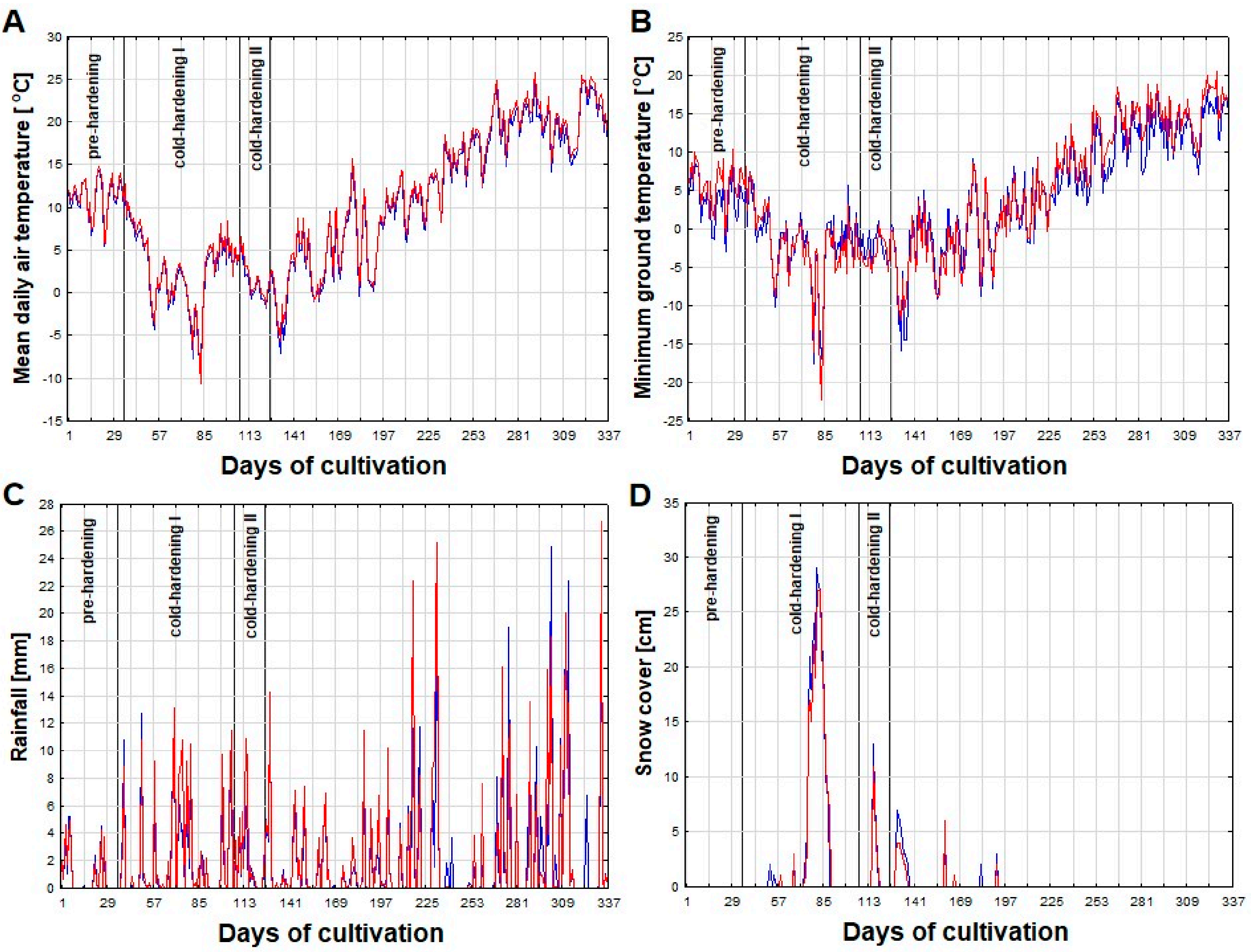

Figure S1. Climatic conditions: A) average daily temperature; B) daily minimum ground temperature; C) daily rainfall and D) daily snow cover depth, measured by the Kraków-Obserwatorium (red line) and Kraków-Balice (blue line) meteorological stations during the winter triticale field cultivation period from September 28, 2022 to August 31, 2023.

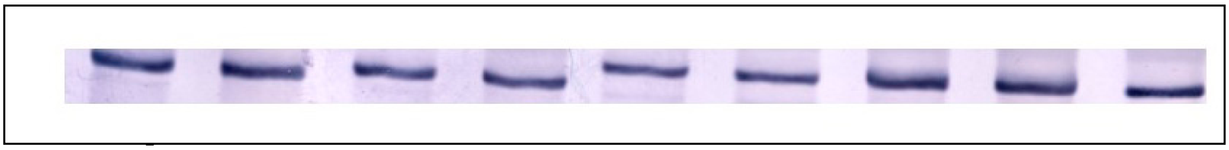

Figure S2. The reference immunostained blot membrane with visible PrxQ bands approx. 28 kDa.
